# Study on the Effect of Additives on the Performance of Cement-Based Composite Anti-Corrosion Coatings for Steel Bars in Prefabricated Construction

**DOI:** 10.3390/ma17091996

**Published:** 2024-04-25

**Authors:** Hao Peng, Zhishan Chen, Mingxiao Liu, Yunlong Zhao, Wanwan Fu, Jiesheng Liu, Xiaoming Tan

**Affiliations:** School of Civil Engineering and Architecture, Wuhan Polytechnic University, Wuhan 430023, China

**Keywords:** polymer cement-based coatings, coated steel, anti-corrosion coatings, corrosion resistance, bonding properties

## Abstract

The influence of polymer emulsion, pigment filler, and dispersant on the corrosion resistance of polymer cement-based composite anti-corrosion coatings were investigated in this study. Adhesion loss rate tests and electrochemical tests were conducted on samples. The research results show that optimal corrosion resistance can be achieved with a 45 wt% dosage of emulsion, a 6 wt% dosage of pigment filler, and a 0.30 wt% dosage of dispersant. The bonding properties of bare steel bars, epoxy-coated steel bars, and polymer cement-based composite anti-corrosion coated steel bars with grout were compared. The results show that the polymer cement-based composite anti-corrosion coating can enhance the bonding properties of the samples. Furthermore, the microscopic analysis was conducted on the samples. The results demonstrate that the appropriate addition of emulsion can fill internal pores of the coating, tightly bonding hydration products with unhydrated cement particles. Moreover, incorporating a suitable dosage of functional additives enhances the stability of the coating system and leads to a denser microstructure.

## 1. Introduction

With the emergence of the green building concept, prefabricated construction is gradually gaining widespread application [1,2]. Compared to conventional building materials, prefabricated structures offer numerous advantages such as shortened construction periods, reduced carbon emissions, and minimized generation of construction waste, thereby promoting environmental friendliness, energy efficiency, and improved productivity [3,4,5]. However, certain challenges persist in the construction process. For example, the steel bar in prefabricated components is susceptible to corrosion during production, storage, transportation, and installation. This not only results in significant economic losses but also destroys the bonding surface with the subsequently poured concrete and undermines the interlocking interaction between the reinforcement and the concrete, thereby leading to potential implications on overall structural load-bearing capacity and performance of buildings [6,7,8]. Therefore, addressing the issue of steel bars corrosion in prefabricated construction is of significant importance.

Based on the current state of research, the protection of steel bars can be approached from two perspectives. First is indirect protection, achieved by enhancing the impermeability of concrete to optimize its pore structure, improving the internal structure’s density, and slowing down the corrosion rate of steel bars by chloride ions and moisture [9,10]. Second is direct protection, which can be achieved by modifying carbon steel through the addition of alloy elements to enhance its inherent corrosion resistance [11,12]. Alternatively, a protective anti-corrosion coating can be applied to the surface of the steel bar, effectively isolating the carbon steel from corrosive media, thereby decelerating the electrochemical corrosion rate and generating a shielding effect [13,14]. Anti-corrosion coatings have been widely used in various construction fields due to their excellent corrosion resistance, easy application, and environmental friendliness. According to chemical composition, it can be divided into three main categories: organic coatings, inorganic coatings, and organic-inorganic composite coatings [15]. Organic coatings refer to coatings composed mainly of polymeric compounds as the film-forming materials, such as epoxy coatings, polyurethane coatings, and acrylic coatings. Inorganic coatings include silicate coatings, phosphate coatings, and inorganic zinc-rich coatings, etc. [16,17,18,19,20,21]. However, organic coatings are prone to issues such as peeling and cracking due to the evaporation of organic solvents which compromises their weather resistance [22,23]. Inorganic coatings, on the other hand, exhibit lower adhesion and ductility due to their higher cleanliness requirements for substrate surfaces and significant susceptibility to construction environment influences [24,25,26]. Polymer cement-based composite anti-corrosion coatings combine polymer emulsions with cement-based materials, incorporating a certain amount of pigment fillers and functional additives [27]. This constitutes an organic-inorganic hybrid material that aims to uphold exceptional adhesion and bonding strength while optimizing its corrosion resistance capabilities.

In recent years, research on polymer cement-based materials in the field of waterproof coatings is extensive. Liang et al. [28] combined silicone-modified polyacrylate (SPA) emulsion and calcium sulfoaluminate (CSA) cement, and determined the optimal polymer to cement ratio (p/c), the dosage of film-forming additive and defoamer through the analyses of the basic physical properties, tensile properties, and water absorption. Compared with the commercial polymer cement-based coatings and organic coatings, the resulting coatings have better alkali resistance, chloride ion penetration resistance and temperature resistance. Li et al. [29] investigated the impact of flake waste glass powder and rutile titanium dioxide (TiO_2_) dosage and ratio on the tensile properties, thixotropy, water absorption, and anti-ultraviolet light (UV) aging resistance of coatings based on the theory of pigment volume concentration (PVC)/critical pigment volume concentration (CPVC). The results showed that when the dosage of pigment filler was 20 wt% and waste glass powder/TiO_2_ was 2:1, the tensile strength of the coatings increased by 49% and the water absorption reduced by 15%, accompanied by the good thixotropy. Lu et al. [30] introduced polydimethylsiloxane (PDMS) into polymer cement-based coatings, and investigated the effects of PDMS hydrophobically modified polyacrylate emulsion on the tensile strength, adhesion, water absorption, UV aging, and salt water immersion resistance. The resulting polymer cement-based coatings showed significant improvement in deterioration and UV aging resistance compared with ordinary coatings.

The above studies indicate that there is relatively limited research on polymer cement-based coatings in the field of steel bar anti-corrosion coatings. Most studies primarily focus on the tensile properties, basic physical properties, and water resistance of the coatings, with very few studies addressing corrosion resistance. Furthermore, there is a lack of detailed research on the bond strength between the coatings and the steel bar as well as the concrete. The objective of this study is to develop a novel composite anti-corrosion coating specifically designed for prefabricated construction steel bars. Building upon the basic formulation of the coating, further research was conducted to investigate the impact of emulsion, pigment filler, and dispersant dosage on the corrosion resistance properties of the coating. To ensure that the coating has a certain degree of corrosion resistance properties without compromising the bond strength with the steel bar and concrete, a comparative analysis was conducted on the failure modes, load-bearing capacity, and deformation capacity of bare steel bars samples, epoxy-coated steel bars samples, and polymer cement-based composite anti-corrosion coated steel bars samples in semi-grouted sleeve steel bar connections. Furthermore, the microscopic morphology of the coatings was observed using a scanning electron microscope (SEM), and the types of hydration products of cement and the composition of functional groups in the coatings were characterized using a Fourier transform infrared spectroscopy (FTIR). The study explored the mechanism of action of emulsions and functional additives on anti-corrosion coatings, providing data support and theoretical basis for subsequent research on polymer cement-based composite anti-corrosion coatings.

## 2. Materials and Methods

### 2.1. Raw Materials

The raw materials for the preparation of polymer cement-based composite anti-corrosion coatings consist primarily of two components: powder materials and liquid materials. The powder materials include complex portland cement (P-C 42.5, Huaxin Cement Factory, Wuhan, China), Ferric oxide (Fe_2_O_3_, McLean Biochemical Technology Co., Ltd., Shanghai, China). Liquid materials include waterborne epoxy resin emulsion (Yoshida Chemical Co., Ltd., Shenzhen, China), PCE-11 polycarboxylic acid water reducing agent (Yusuo Chemical Co., Ltd., Linyi, China), Ecowet OX-4070 wetting and dispersing agent (Yusuo Chemical Co., Ltd., Linyi, China), film-forming additives (Alcohol ester XII, Yoshida Chemical Co., Ltd., Shenzhen, China), defoamer (Mengtai Weiye Building Materials Co., Ltd., Beijing, China).

The materials used for the preparation of the semi-grouted sleeve steel bar connections specimens included HRB400 threaded steel bars (diameter 14 mm), cementitious dry mix consisting of special cement, fine aggregates, and a variety of functional admixtures (Pintai Special Building Materials Technology Co., Ltd., Wuhan, China).

### 2.2. Instrumentation

The main instruments and equipment used in the preparation and performance testing of the coatings in this experiment are presented in Table 1.

### 2.3. Mixing Ratio Design

In the preceding period, we conducted a substantial number of exploratory experiments to systematically investigate the impact of each component on the fundamental physical properties of the material. Ultimately, we concluded that optimal comprehensive physical properties for the coating formulation were achieved by employing a polymer emulsion dosage of 45 wt%, pigment filler dosage of 6 wt%, dispersant dosage of 0.30 wt%, and defoamer dosage of 0.20 wt%. This formulation was designated as basic formulation L1.

Building upon the basic formulation L1, and keeping other conditions constant, changing the dosage of polymer emulsions (30 wt%, 35 wt%, 40 wt%, 45 wt%, 50 wt%) to determine the optimal amount of emulsion dosing based on the fundamental properties and corrosion resistance test results of the coatings produced with different emulsion dosage. Upon identifying the optimal emulsion dosage, a controlled variable approach was employed to systematically investigate the influence of pigment filler (0 wt%, 2 wt%, 4 wt%, 6 wt%, 8 wt%, 10 wt%) and dispersant (0 wt%, 0.20 wt%, 0.30 wt%, 0.40 wt%, 0.60 wt%, 0.80 wt%) on material performance, ultimately determining the optimal dosage for each raw material. The mass ratio of all components is based on cement. The mixing ratio design is shown in Table 2.

### 2.4. Preparation of Samples and Coated Test Panels

#### 2.4.1. Coating and Performance Test Panel Preparation

Firstly, weigh the waterborne epoxy resin emulsion, curing agent, deionized water and functional additives according to the experimental formulation. Using an electric stirrer stirred at 500 rpm for 2 min to obtain a uniformly mixed liquid. Subsequently, weigh the complex portland cement after 50-mesh screen, pigment filler, manually stirred for 1 min to achieve a uniformly mixed powder. Finally, blend the powder into the liquid, stirred at 700 rpm for 6 min. After stirring ceased, stand the mixture for 1 min, resulting in the polymer cement-based composite anti-corrosion coating.

**Figure 1:**
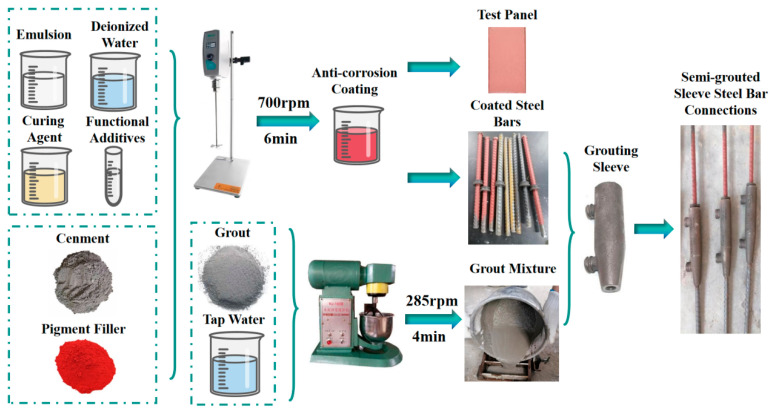
Samples and coated test panels preparation process.

The tinplates were processed according to Chinese national standard GB/T 9271 [31]. Subsequently, the prepared coating was applied to the surface of the tinplates with KTQ-III adjustable film applicator, and the film thickness was fixed at 1 mm. The coated plates were cured under constant temperature and humidity conditions (temperature 23 ± 2 °C, relative humidity 50% ± 5%) for 48 h to obtain the test panel of the polymer cement-based composite anti-corrosion coating. The preparation process is illustrated in Figure 1.

#### 2.4.2. Preparation of Samples for Semi-Grouted Sleeve Steel Bar Connections

The specific steps for preparing the semi-grouted sleeve steel bar connections samples are as follows:

(1) Use a brush to clean the surface of the steel bars and the interior of the sleeve, removing dust and rust, preparing epoxy-coated steel bars and polymer cement-based composite anti-corrosion coated steel bars.
(2) Connect the steel bars at both ends of the grouting sleeve, using threaded connection at one end and fixing the other end with a sealed rubber ring. The anchoring depth of the steel bars is set at 115 mm. After completing the steel bar connection, secure the semi-grouted sleeve with connected steel bars to the iron frame using nylon straps.
(3) According to the ratio of grout dry material to mixing water (100:15), weigh the grout and tap water. Transfer the grouting dry materials into a mixing pot and mix for 1 min, gradually incorporating the water during this process, and then mix for an additional 4 min. After thorough mixing, allow it to stand undisturbed for 2 min.
(4) Pour the prepared grout mixture into a manual grouting device, adhering to the principle of “low in, high out”. Inject the grout through the bottom grouting orifice until it emerges from the upper discharge orifice. Subsequently, seal both the grouting and discharge orifices and allow for undisturbed curing of the sample for a duration of 72 h.

### 2.5. Testing and Characterization

#### 2.5.1. Adhesion Loss Rate Test

The prepared coating test panels will be cured under standard conditions for 48 h. According to the Chinese national standard GB/T 5210-2006 [32], the adhesion strength of the coating will be measured at this point and recorded as *F*_0_. Subsequently, the test panels will be immersed in deionized water. After soaking for 7 days, the panels will be removed, excess surface moisture will be absorbed using filter paper, and the adhesion strength of the panels after immersion will be measured and recorded as *F*_1_. Each group of samples undergoes three tests, and the average value is taken from the valid data.

The formula for calculating the coating’s adhesion loss rate is as follows:

(1) $$L_{F}=\frac{\left(F_{0}-F_{1}\right)}{F_{0}}\times 100\%$$

where: *L_F_* is the adhesion loss rate of the coating after soaking for 7 days (%), *F*_0_ is the adhesion strength measured before immersion (MPa), *F*_1_ is the adhesion strength measured for the same coating test panel after soaking for 7 days (MPa).

#### 2.5.2. Electrochemical Test

The polished tinplate and test panels were employed as the working electrodes (with a working area of 1 cm^2^), aligned with the test holes on the flat corrosion cell, and the bolts were tightened to ensure a tight seal between the working electrodes and the PTFE face of the corrosion cell. Subsequently, a 3.5% NaCl solution was injected into the corrosion cell, left to stand for 1 min, and the density of the corrosion cell was checked for any solution leakage at the test hole locations. Finally, a saturated calomel electrode (SCE, reference electrode) was inserted into the flat plate corrosion cell, connected to the wires, and electrochemical testing was conducted. Initially, open circuit potential (OCP) tests were performed on the samples with a testing time of 30 min. Upon stabilization of the OCP, sequential tests including potentiodynamic polarization tests and electrochemical impedance spectroscopy (EIS) were carried out. For EIS, an alternating current amplitude of 10 mV and a scan frequency range of 0.01 Hz to 100 KHz was employed. All the tests were performed in triplicate.

#### 2.5.3. Bonding Properties Test

The semi-grouted sleeve steel bar connection samples were vertically fixed on the universal testing machine, and incremental loads were applied from zero until the samples failed. After completing the tests, the load-displacement curves for each sample were exported. Subsequently, the yield strength, tensile strength, and bond strength of the samples were calculated using the Formulas (2), (3) and (4), respectively.

(2) $$R_{e}=\frac{F_{e}}{S_{0}}$$

where:$R_{e}$ is the yield strength of the sample in megapascals (MPa); $F_{e}$ is the yield load of the sample in kilonewtons (KN); $S_{0}$ is the cross-sectional area of the steel bar, which is 153.9 mm^2^.

(3) $$R_{m}=\frac{F_{b}}{S_{0}}$$

where: $R_{m}$ is the tensile strength of the sample in megapascals (MPa); $F_{b}$ is the ultimate failure load of the sample in kilonewtons (KN); $S_{0}$ is the cross-sectional area of the steel bar, which is 153.9 mm^2^.

(4) $$\tau =\frac{F_{b}}{\pi dl}$$

where: τ is the bond strength of the sample in megapascals (MPa); $F_{b}$ is the ultimate failure load of the sample in kilonewton, (KN); d is the nominal diameter of the steel bar, which is 14 mm, l is the embedded length of the steel bar, which is 115 mm.

#### 2.5.4. SEM Analysis

The prepared coating was poured into molds and cured for the specified aging period. Afterward, it was cut into 0.5 cm × 0.5 cm square samples, affixed to conductive adhesive, and sputter-coated with gold for 45 s at 10 mA using the Oxford Quorum SC7620 sputter coater (Quorum Technologies Ltd., East Sussex, UK). Subsequently, the cross-sectional morphology of the coating was captured at 2000 times magnification using a ZEISS (Carl Zeiss) SEM.

#### 2.5.5. FTIR Analysis

The prepared coating was poured into molds and cured for the specified aging period. After removal, it was dried and ground into powder using a mortar and pestle, followed by sieving through a 100-mesh screen. Approximately 1–2 mg of the powdered sample was mixed with 200 mg of pure potassium bromide (KBr), thoroughly ground to ensure uniformity, and pressed into a transparent sheet using a hydraulic press. The resulting sheet was then subjected to infrared spectrometer in the range of 4000~600 cm⁻^1^ with 32 scans and a resolution of 4 cm⁻^1^.

## 3. Results and Discussion

### 3.1. Effect of Different Raw Material Dosing on Coating Adhesion

**Figure 2:**
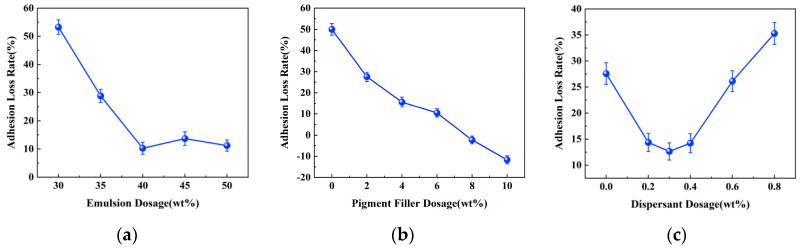
Effect of different raw material dosage on the rate of coating adhesion loss. (**a**) Effect of emulsion dosage. (**b**) Effect of pigment filler dosage. (**c**) Effect of dispersant dosage.

The adhesion strength of the coating refers to the force required to peel the coating vertically from the substrate surface and is a crucial indicator for assessing the adhesion between the coating and the substrate [33,34]. Adhesion strength tests were conducted on coated test panels with different dosages of raw material before and after immersion. The adhesion loss rate was calculated, which can judge the denseness and water resistance of the coating, and thus the protective effect of the coating on the metal substrate. The test results are presented in Figure 2.

From the graph, it is evident that the adhesion strength of the coatings produced with different raw material dosages all experienced a decrease after immersion due to incomplete densification resulting from existing pore spaces within the coating. During immersion, water molecules penetrate the coating through these pores, displacing the coating-substrate interface and compromising bonding between them, ultimately leading to reduced adhesion strength.

The impact of the emulsion dosage on the adhesion strength loss rate of the coating is depicted in Figure 2a. It can be observed that as the polymer emulsion dosage increases, there is initially a significant decrease in the rate of adhesion strength loss, followed by a gradual stabilization trend. When the emulsion dosage reaches 40 wt%, the coating’s adhesion strength reaches its minimum at 10.25%. This phenomenon can be attributed to the inherently excellent adhesion performance of the water-based epoxy resin. Furthermore, after uniform mixing with the cement slurry, the film-like substance formed by the curing of epoxy resin can enhance the interaction with cement. It intertwines with the hydration products to create a 3D network structure, effectively filling the voids between the cement hydration products [35,36]. This improves coating density and provides resistance against water molecules while enhancing substrate surface erosion prevention capabilities.

The impact of pigment filler dosage on the adhesion strength loss rate of the coating is illustrated in Figure 2b. It can be observed that as the pigment filler dosage increases, there is a continuous decrease in the rate of adhesion strength loss for the coating. Moreover, at pigment filler dosages of 8 wt% and 10 wt%, the adhesion strength of the coating increases after immersion for 7 days, resulting in a negative adhesion strength loss rate. This phenomenon can be attributed to the effective filling of minuscule gaps between coating layers by the pigment filler, thereby enhancing the compactness of the coating. Additionally, the pigment filler reduces the surface tension of the coating, facilitating its spread over the substrate and thereby increasing adhesion strength. The abnormal phenomenon of increased adhesion strength after immersion may be attributed to cement-based nature of this particular coating material. Immersion likely provides a certain curing effect on the coating, slowing down the degradation process and improving the adhesion performance of the coating.

The impact of the dispersant dosage on the adhesion strength loss rate of the coating is depicted in Figure 2c. It can be observed that as the dispersant dosage increases, initially there is a decrease followed by an increase in the adhesion strength loss rate of the coating. The minimum adhesion strength loss rate is observed at a dispersant dosage of 0.30 wt%. This phenomenon can be attributed to the fact that an appropriate amount of dispersant can enhance the dispersion of fillers, preventing the agglomeration and settling of filler particles, improving the stability of the coating system [37]. This results in a smoother and denser coating with positive effects on adhesion strength. However, excessive dispersant addition may reduce the stability of the coating system, affecting viscosity, coating amount, storage performance, and construction properties. This makes the coating more susceptible to water penetration, leading to a faster rate of adhesion strength loss.

### 3.2. Electrochemical Test

(1) Tafel Polarization Curve Test

**Figure 3:**
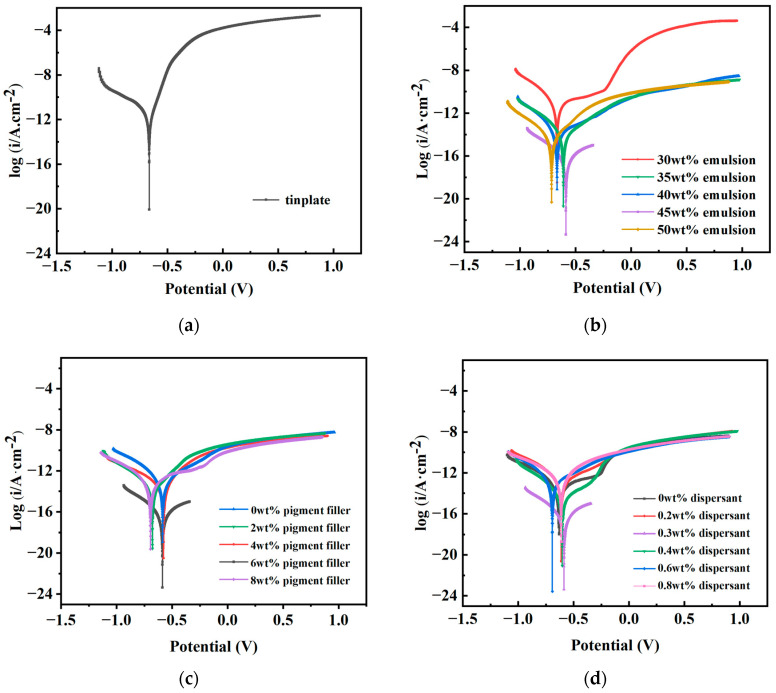
Polarization curves of coated test plates made of tinplate and different raw material dosages. (**a**) Polarization curve of tinplate. (**b**) Polarization curve of different emulsion dosages. (**c**) Polarization curve of different pigment filler dosages. (**d**) Polarization curve of different dispersant dosages.

The Tafel polarization curve test is conducted to evaluate the corrosion resistance of materials. By utilizing CSstudio5 software (5.4.627.16) for fitting, the material’s corrosion potential (E_corr_) and corrosion current density (i_corr_) can be directly determined, thereby enabling a direct assessment of the material’s corrosion resistance [38]. Typically, the primary parameter of interest is i_corr_; the smaller the i_corr_, the slower the material’s corrosion rate, indicating superior corrosion resistance. When the i_corr_ values of two materials are roughly equivalent, E_corr_ becomes a parameter of consideration, a more positive E_corr_ indicates better corrosion resistance. Polarization curve tests were conducted on coated test panels made from different raw materials, and the test data, as shown in Figure 3, yielded the fitted E_corr_ and i_corr_ results as presented in Table 3.

From Table 3, it is evident that the i_corr_ of the uncoated tinplate is significantly higher than that of the coated counterparts, indicating that the anti-corrosion coatings effectively inhibit the ingress of corrosive media, thereby retarding the corrosion rate and providing notable protective effects to the substrate. Furthermore, Table 3 reveals that at a dispersant dosage of 0.3 wt% (D3), the coated layer exhibits an E_corr_ of −0.587 V and an i_corr_ of 2.72 × 10^−7^ A·cm^−2^, which represent the maximum potential and the minimum current among the six different dosages. It is noteworthy that while D3 demonstrates a marginal advantage in terms of E_corr_ compared to other formulations, it exhibits a significant advantage in i_corr_, being one order of magnitude lower than the next smallest value (D4), indicating that at a dispersant dosage of 0.3 wt%, the permeability of corrosion ions is minimal, thereby imparting optimal corrosion resistance to the coating.

In the fitted values of different dosages of pigment fillers, it can be observed that P1, P2, P3, P4, and P5 exhibit approximately the same E_corr_, whereas the i_corr_ of P4 is significantly lower than that of the other formulations. This phenomenon arises because the pigment fillers, when combined with water, provide the coating with enhanced protective properties. Insufficient pigment fillers cannot fully resist corrosion caused by the original battery reaction, while excessive pigment fillers increase the viscosity of the coating, leading to aggregation and reduced dispersibility. Similarly, in the fitted values of different emulsion dosages, E4 demonstrates a similar phenomenon as described above. When the emulsion dosage is 45 wt%, the i_corr_ of the coating is minimized, resulting in improved electron barrier effects. Excessive emulsion addition increases coating viscosity, leading to increased bubbles and affecting coating stability, while insufficient emulsion addition results in unfilled pores formed during cement hydration, affecting coating density.

In conclusion, comprehensive analysis suggests that when the emulsion, pigment filler, and dispersant dosages are 45 wt%, 6 wt%, and 0.30 wt%, respectively, the maximum protection of the substrate and enhanced corrosion resistance of the coating can be achieved.

(2) EIS test

**Figure 4:**
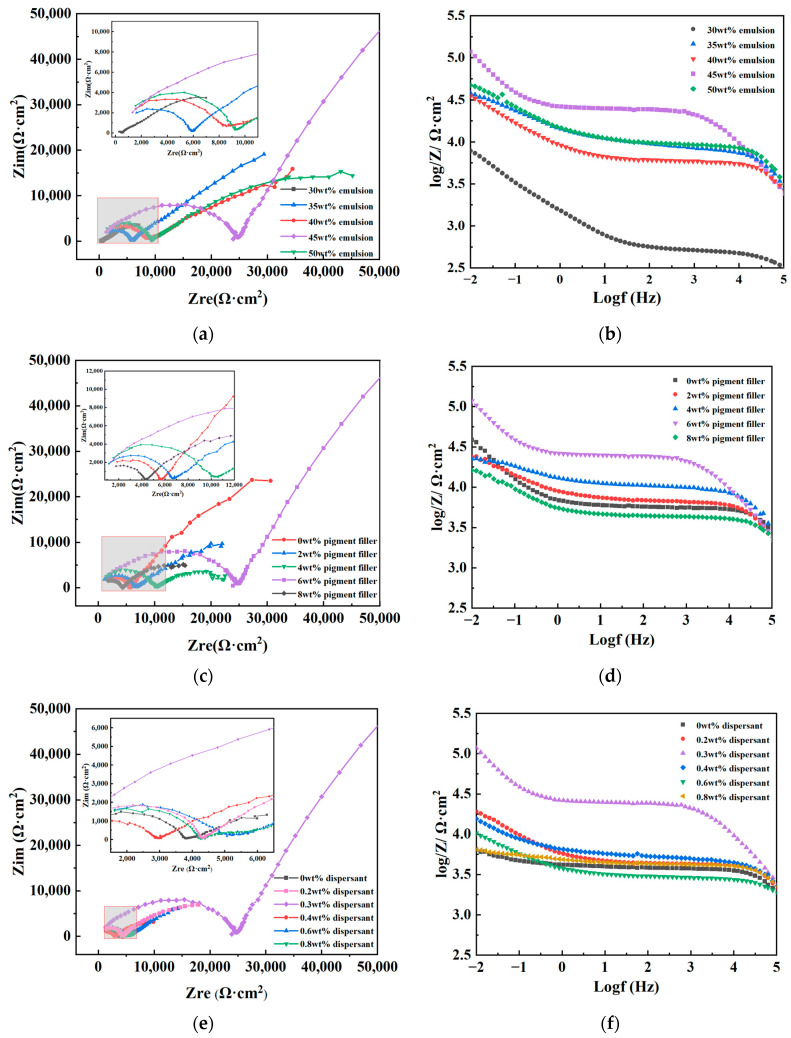
Nyquist plot and Bode plot of coated test plates made of different raw material dosages. (**a**) Nyquist plot of different emulsion dosages. (**b**) Bode plot of different emulsion dosages. (**c**) Nyquist plot of different pigment filler dosages. (**d**) Bode plot of different pigment filler dosages. (**e**) Nyquist plot of different dispersant dosages. (**f**) Bode plot of different dispersant dosages.

EIS is a curve plotted from impedance data measured at different frequencies for a test circuit. There are various types of EIS, with the most commonly used being the Nyquist plot and the Bode plot. In a Nyquist plot, the size of the impedance radius is typically utilized to assess the corrosion resistance performance, where a larger impedance radius indicates better corrosion resistance of the material. In the Bode plot, the impedance modulus at 0.01 Hz is often employed to describe the shielding performance of coatings, with a higher value indicating a stronger ability of the coating to resist external corrosion [39,40,41]. Impedance spectroscopy tests were conducted on coated test panels made from different raw materials, and the test data are illustrated in Figure 4.

From Figure 4a,c,e, it is evident that the Nyquist plots of coatings made from different raw material dosages exhibit a characteristic of double capacitive arcs, including high-frequency and low-frequency capacitive arcs, indicating reactions between the corrosive medium and the substrate through the coating during the corrosion process. The high-frequency capacitive arc reflects the difficulty of electron transfer between the coating and the corrosive medium. It can be observed that with an increase in the dosage of each raw material, the impedance radius generally follows a trend of initially increasing and then decreasing. This indicates that the addition of each component in either small or excessive amounts can affect the anti-corrosion effectiveness of the coating. When the emulsion, pigment filler, and dispersant dosages are 45 wt%, 6 wt%, and 0.30 wt%, respectively, the impedance radius is significantly greater than that of other formulations, indicating that electron transfer between the coating and the corrosive medium is most difficult under this condition, making corrosion reactions relatively more challenging compared to other formulations.

In Figure 4b,d,f, the Bode plots all display two peaks, indicating two time constants during the corrosion process. The low-frequency peak reflects the penetrability of the corrosive medium. It can be observed that when the emulsion, pigment filler, and dispersant dosages are 45 wt%, 6 wt%, and 0.30 wt%, respectively, the impedance modulus at 0.01 Hz reaches a maximum value of 1.18 × 10^5^ Ω·cm^2^, which is one order of magnitude higher than the poorest-performing group. This suggests that the coating formulation exhibits optimal shielding capability against the corrosive medium and demonstrates the best corrosion resistance.

**Figure 5:**
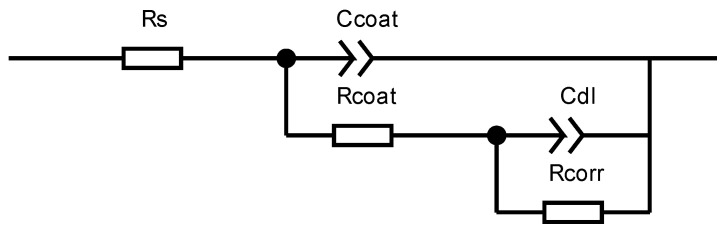
Equivalent circuit used to fit the EIS results.

In order to provide a clearer reflection of the corrosion status of the coatings, impedance spectra were fitted using CSstudio5 software, employing an equivalent circuit as illustrated in Figure 5. Herein, Ccoat represents the capacitance of the coating itself, Rcoat denotes the resistance of the coating, Cdl signifies the double-layer capacitance at the metal surface beneath the coating, and Rcorr represents the impedance of the electrode reaction when the solution permeates the coating and reacts electrochemically at the metal surface. The fitted value of Rcoat is presented in Table 4.

From the fitting results, it can be observed that when the emulsion, pigment filler, and dispersant dosages are 45 wt%, 6 wt%, and 0.30 wt%, respectively, the resistance of the coating, Rcoat, can reach a maximum value of 2.49 × 10^4^ Ω·cm^2^, significantly higher than that of other formulations. Based on the comprehensive analysis of EIS test results, fitting results, and polarization curve test results, it can be concluded that when the emulsion, pigment filler, and dispersant dosages are 45 wt%, 6 wt%, and 0.30 wt%, respectively, the corrosion resistance of the coating is optimal. Both excessive and insufficient dosages lead to performance degradation.

### 3.3. Bonding Properties Test

Based on the combined results of adhesion loss rate and electrochemical tests, it can be concluded that the coating formulation L2 with the optimal corrosion resistance performance is consistent with the base formulation L1. The semi-grouted sleeve steel bar connection samples made of bare steel bars, epoxy-coated steel bars, and polymer cement-based composite anti-corrosion coated steel bars are designated as N_0_, N_1_, and N_2_, respectively. Labels are affixed to the sleeves for identification.

#### 3.3.1. Sample Damage Pattern

**Figure 6:**
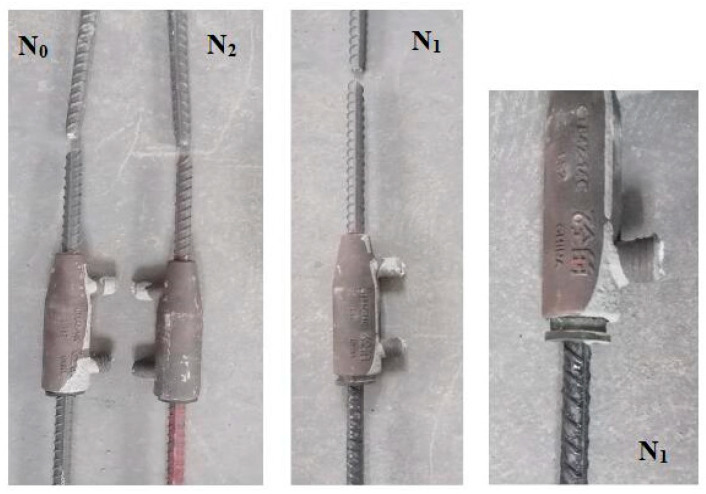
Damage morphology of semi-grouted sleeve steel bar connection samples.

The damage morphology of each semi-grouted sleeve steel bar connection sample is illustrated in Figure 6. It can be observed that connection samples N_0_, N_1_, and N_2_, prepared using bare steel bars, epoxy-coated steel bars, and polymer cement-based composite anti-corrosion coated steel bars, respectively, all experienced failure after unidirectional tensile testing. In each sample, the threaded connection end of the steel bar underwent tensile fracture. Additionally, in sample N_1_, the steel bar at the rubber ring fixing end was pulled out.

#### 3.3.2. Sample Unidirectional Tensile Test Results

**Figure 7:**
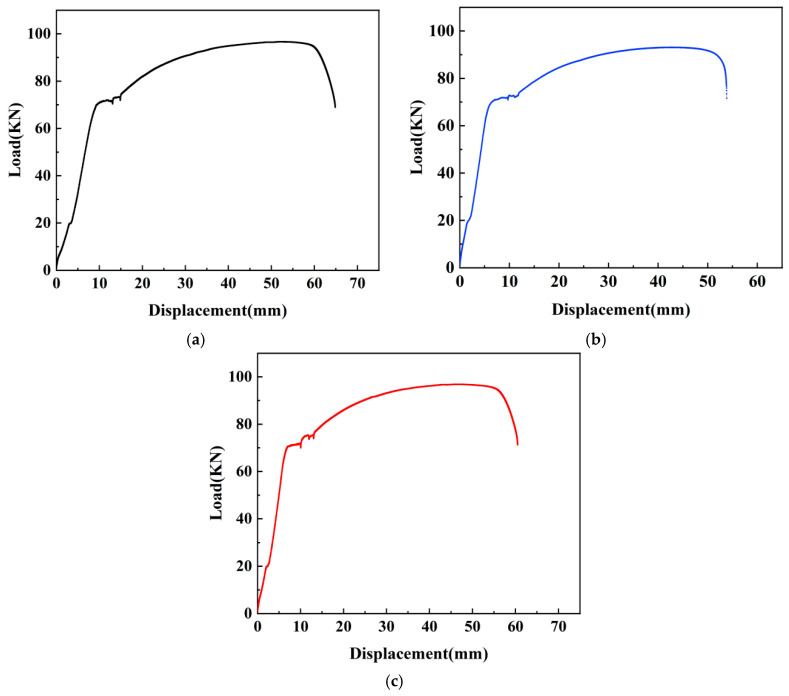
Load-displacement curves of samples N_0_, N_1_ and N_2_. (**a**) Load-displacement curve of N_0_. (**b**) Load-displacement curve of N_1_. (**c**) Load-displacement curve of N_2_.

Unidirectional tensile tests were conducted on samples N_0_, N_1_, N_2_, and their corresponding load-displacement curves are presented in Figure 7, and the specific data of tensile tests were shown in Table 5.

Upon observing the load-displacement curves of each sample, it becomes evident that the overall pattern of change in each curve is quite similar, which can be divided into four stages: the first stage (elasticity stage), belongs to the initial loading phase where there exists an approximately linear relationship between load and displacement; as displacement increases, so does the load. The second stage (yielding stage), the yielding plateau occurs, with slight fluctuations in load and instances of displacement increase without significant load changes. The third stage (strengthening stage), both displacement and load of the sample continue to increase, but the curvature of the curve gradually decreases, approaching a horizontal position. The load gradually reaches its limit. The fourth stage (necking stage), noticeable thinning occurs on the sleeve connected to the steel bar, displaying necking behavior, the load decreases in a linear manner, and the steel bar is pulled off.

Upon observing Table 5, it is evident that the measured values of yield strength, tensile strength, and bond strength for each sample exhibit a remarkable proximity. For sample N_0_, the yield strength is 457.40 MPa, the tensile strength is 628.07 MPa, and the bond strength is 19.11 MPa. Sample N_1_ has a yield strength of 465.35 MPa, representing a 1.74% increase compared to N_0_, while the tensile strength and bond strength are 604.74 MPa and 18.40 MPa, indicating a 3.72% decrease from N_0_. Sample N_2_ exhibits a yield strength of 478.74 MPa, marking increases of 4.67% and 2.88% compared to N_0_ and N_1_, respectively. The tensile strength and bond strength for N_2_ are 629.37 MPa and 19.15 MPa, showing increases of 0.21% and 4.08% compared to N_0_ and N_1_, respectively.

The test results indicate that the epoxy resin coating adversely affects the bond between the steel bar and grout, resulting in a reduction in the load-bearing capacity of the samples. Conversely, the application of polymer cement-based composite anti-corrosion coating does not significantly impact the load-bearing capacity and deformation capacity of the samples. In fact, it may even enhance to some extent the bond anchorage between the steel bar and grout.

### 3.4. SEM Analysis

(1) Mechanism of polymer emulsion on anti-corrosion coating

In order to investigate the mechanism of polymer emulsion on anti-corrosion coatings, SEM images were taken for coatings prepared with 30 wt% emulsion, 45 wt% emulsion, and 50 wt% emulsion. The cross-sectional morphology and compactness of the coatings were observed.

**Figure 8:**
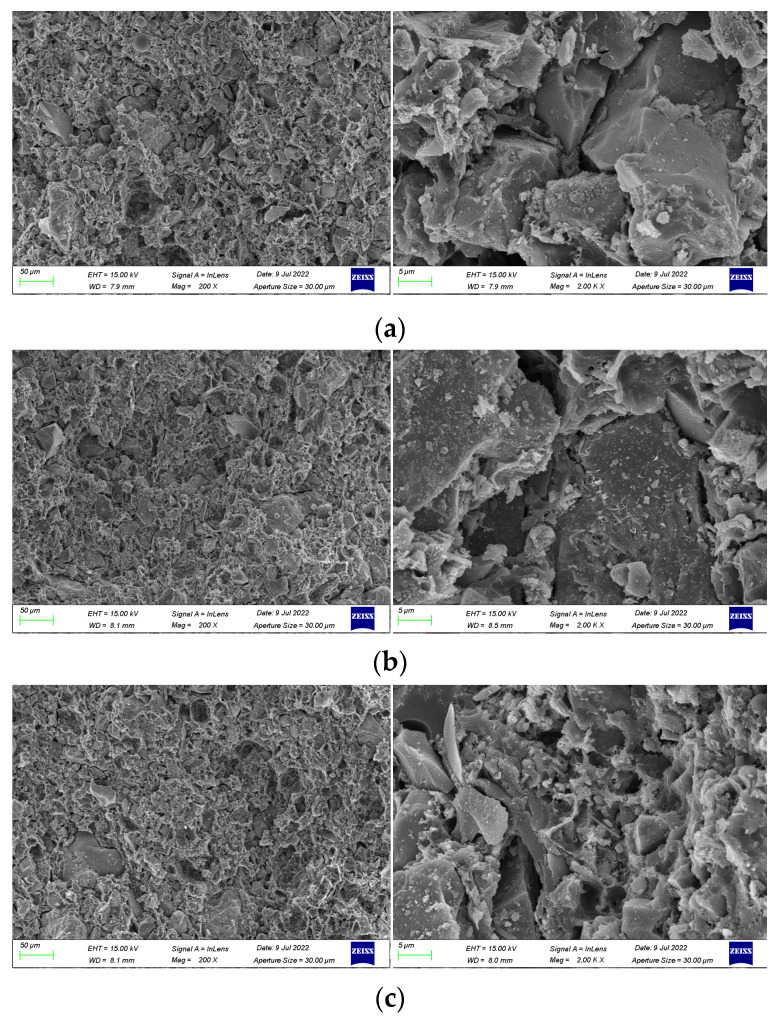
SEM images of coating samples made with different emulsion dosages. (**a**) 30 wt% emulsion. (**b**) 45 wt% emulsion. (**c**) 50 wt% emulsion.

SEM images magnified 200 times and 2000 times of coatings prepared with varying emulsion dosages are presented in Figure 8. The observations from the figure reveal that the inclusion of polymer emulsion has a certain impact on the formation of cement hydration products. As shown in Figure 8a, when the emulsion dosage is 30 wt%, the coating is primarily composed of cement hydration products, with a relatively small proportion of waterborne epoxy resin. Additionally, the polymer emulsion fails to completely fill the voids in the hydration products, leading to a comparatively loose internal structure of the coating, numerous pores on the cross-section, and a relatively low cross-sectional smoothness.

From Figure 8b, it can be observed that a significant portion of the epoxy resin has cured into an amorphous film, with some of it filling the voids generated by cement hydration and the rest covering the non-hydrated cement particles. This enhances the compatibility between cement hydration products and cement particles, resulting in a denser internal structure of the coating and enhanced coating performance [42,43,44].

As depicted in Figure 8c, it is evident that the polymer emulsion tightly envelops the cement particles and continues to solidify into a film-like substance, resulting in an increased thickness of the cement hydration layer. This impedes the cohesion between cement particles, slowing down the rate of cement hydration and hindering the hydration process. Moreover, due to the high viscosity of epoxy resin, air bubbles are introduced during agitation, leading to the presence of numerous pores on the surface of the coating and a more porous internal structure [45].

(2) Mechanism of functional additives on anti-corrosion coating

**Figure 9:**
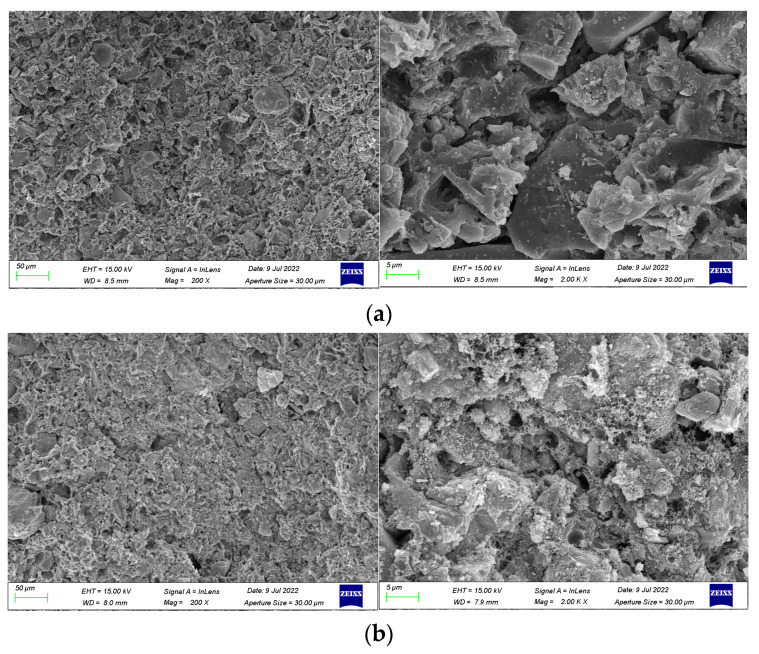
SEM image of blank coating samples without doping of pigment filler and dispersant.: (**a**) without pigment filler and (**b**) without dispersant.

To elucidate the underlying mechanism of functional additives in anti-corrosion coatings, SEM images were taken of blank coatings without pigment filler and without dispersant. These were subsequently compared with the SEM image Figure 8b of the optimally formulated coating. The SEM images of the coating samples are presented in Figure 9.

From Figure 9a, it is observed that the coating contains uncured spherical epoxy resin particles and exhibits particle aggregation in the emulsion phase. This phenomenon can be attributed to the absence of pigment filler, resulting in a lower solid content and a relatively higher proportion of undispersed epoxy resin within the coating. This also leads to a decrease in the viscosity of the coating, disrupting the system stability. In Figure 9b, particle-like hydrated calcium silicate gel (C-S-H), plate-like crystals of calcium hydroxide (Ca(OH)_2_), and needle-like ettringite (AFt) are evident. The absence of a dispersant clearly diminishes the dispersion of cement particles and pigment filler particles in the liquid, and some cement particles undergo hydration reactions, resulting in an enlargement of pores within the coating layer.

### 3.5. FTIR Analysis

Different chemical bonds or functional groups have different absorption frequencies and are in different positions on the infrared spectral map. Therefore, the composition of functional groups in coatings can be determined using an infrared spectrometer. This provides further insights into the influence of polymer emulsion on the cement hydration process and the resulting hydration products.

**Figure 10:**
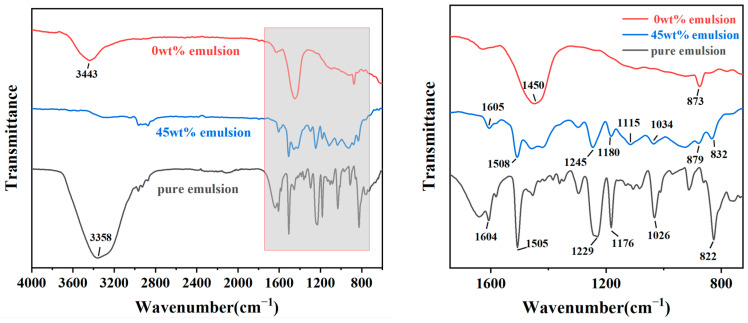
Infrared spectra of coating samples made with 0 wt% emulsion content, optimal emulsion content, and pure epoxy resin emulsion.

FTIR analysis was conducted on the coating samples with 0 wt% emulsion content, optimal emulsion content, and pure epoxy resin emulsion, resulting in FTIR spectra for the polymer cement-based composite anti-corrosion coatings and pure epoxy resin emulsion (Figure 10). The specific analysis is as follows:

(1) The peaks observed in the range of 3358 cm^−1^ to 3443 cm^−1^ are attributed to the stretching vibration peaks of O-H bonds, mainly caused by residual water molecules in the samples. Pure emulsion contains more water, thus exhibiting a significantly higher peak intensity compared to the other two samples. The intensity of the peak in the coating with 0 wt% emulsion content is also higher than that with 45 wt% emulsion content. This phenomenon can be ascribed to the enhanced compactness of the coating after the epoxy resin is incorporated into the cement-based coating, as the curing of epoxy resin forms a film, thereby reducing the ingress of moisture from the air and consequently decreasing the internal moisture content of the coating.
(2) Peaks near 1605 cm^−1^ and 1508 cm^−1^ are mainly characteristic absorption peaks of the benzene ring framework. It is observed that with an increase in epoxy resin content, the intensity of these peaks also increases significantly.
(3) The peak at 1450 cm^−1^ corresponds to the stretching vibration of the C-O bond, representing a characteristic peak indicative of carbonate ions. This can be attributed to the sample being exposed to the air, where the Ca(OH)_2_ present in it absorbs CO_2_ from the air and undergoes a reaction [46]. The intensity of this peak in the coating with 0 wt% emulsion content is significantly higher than that in the coating with 45 wt% emulsion content. This may indicate that the structure of the coating produced without emulsion is more porous, with a higher porosity, and the hydroxides within the cement paste are more likely to come into contact with air and thus react. Therefore, it can be concluded that an appropriate amount of emulsion can densify the internal structure of the coating, consistent with the results of macroscopic performance and microscopic structure tests.
(4) Peaks observed near 1245 cm^−1^ and 1180 cm^−1^ are typically attributed to the C-O absorption peaks of alcohols or phenols [47], while the peak near 1026 cm^−1^ corresponds to the stretching vibration of aromatic hydrocarbons in the main chain of epoxy resin [48]. It can be observed that the peak intensity at these peaks in pure epoxy resin is significantly higher than that in the coating with 45 wt% emulsion content.
(5) The peak near 873 cm^−1^ corresponds to the Al-OH stretching vibration of the [Al(OH)_6_]^3-^ group, representing the characteristic peak of the hydrated product AFt. A higher content of AFt indicates a higher degree of cement hydration. It is noted that after increasing the epoxy resin content from 0 wt% to 45 wt%, the intensity of this peak significantly decreases, indicating that the addition of epoxy resin hinders the hydration process of the cementitious material, consistent with the results of SEM tests.

## 4. Conclusions

The application of anti-corrosion coatings provides an effective solution to combat corrosion in steel bars. However, existing coatings often suffer from problems such as peeling and poor adhesion. In response to these problems, a novel composite anti-corrosion coating specifically designed for prefabricated construction steel bars has been developed. This study investigated the influence of raw materials such as polymer emulsion, pigment filler, functional additives, and their respective dosages on the corrosion resistance and microstructure of the coating. The following conclusions were drawn:

(1) After 7 days of immersion in water, all coatings prepared with different dosages of raw materials exhibited some degree of adhesion loss. Specifically, with the addition of polymer emulsion, the adhesion loss rate initially decreased before leveling off. The addition of pigment filler led to a continuous reduction in the adhesion loss rate, reaching negative values at dosage of 8 wt% and 10 wt%. The incorporation of additives resulted in an initial decrease followed by an increase in the adhesion loss rate.
(2) The coating formulated with a polymer emulsion dosage of 45 wt%, pigment filler dosage of 6 wt%, and dispersant dosage of 0.30 wt% exhibited the smallest measured i_corr_, the largest impedance spectrum radius, the highest resistance, and the best corrosion resistance. Both insufficient and excessive additions can compromise the coating’s density, leading to a reduction in corrosion resistance.
(3) The application of epoxy resin coating reduces the bond strength between the steel bar and the grouting material, leading to a decrease in the load-bearing capacity of the samples. In contrast, the coating with polymer cement-based composite anti-corrosion material, to a certain extent, enhances the frictional resistance between the steel bar and the grouting material, resulting in improved mechanical performance of the samples and a reduced likelihood of slippage at the anchorage end of the steel bar.
(4) The addition of an appropriate amount of emulsion and pigment filler serves to fill the internal pores of the coating, resulting in a denser internal structure. The incorporation of a dispersant ensures a more uniform dispersion of powder in the liquid, preventing flocculation and settling. The combined action of multiple raw materials contributes to a smoother and denser coating, enhancing overall stability and performance.

## Figures and Tables

**Table 1:** Test apparatus and equipment.

Equipment Name	Model Specification	Manufacturer (of a Product)
Digital Timing Stirrer	OA2000plus	Ouhe Machinery Equipment Co., Ltd., Shanghai, China
Electronic Analytical Balance	BSA Series	Sartorius Scientific Instruments Co., Ltd., Beijing, China
Constant Temperature and Humidity Standard Curing Box	HBY-60B	Luda Experimental Instrument Co., Ltd., Shanghai, China
Adjustable Film Applicator	KTQ-III	Xinyi Laboratory Equipment Co., Ltd., Guangzhou, China
Adhesion Tester	OU4060	Oupu Testing Instrument Co., Ltd., Cangzhou, China
Electrochemical Workstation	CS Series	Corrtest Instruments Co., Ltd., Wuhan, China
Universal Testing Machine	HUT Series	Wance Testing Machine Co., Ltd., Wuhan, China
SEM	ZEISS Gemini 300	Carl Zeiss Management Co., Ltd., Shanghai, China
FTIR	ALPHA II	Kinesis Technology Co., Ltd., Wuhan, China

**Table 2:** Design scheme for mixing ratios.

Number	Emulsion (wt%)	Pigment Filler (wt%)	Dispersant (wt%)
E1	30	6	0.30
E2	35	6	0.30
E3	40	6	0.30
E4	45	6	0.30
E5	50	6	0.30
P1	45	0	0.30
P2	45	2	0.30
P3	45	4	0.30
P4	45	6	0.30
P5	45	8	0.30
P6	45	10	0.30
D1	45	6	0.00
D2	45	6	0.20
D3	45	6	0.30
D4	45	6	0.40
D5	45	6	0.60
D6	45	6	0.80

**Table 3:** Fitting results of polarization curves of coated test plates made of tinplate and different raw material dosages.

Sample		Corrosion Potential (vs. SCE)/V	Corrosion Current Density/(A·cm^−2^)
Tinplate		−0.664	7.07 × 10^−5^
Emulsion	E1	−0.258	7.02 × 10^−7^
	E2	−0.614	1.01 × 10^−5^
	E3	−0.667	1.96 × 10^−6^
	E4	−0.587	2.72 × 10^−7^
	E5	−0.716	5.73 × 10^−6^
Pigment filler	P1	−0.615	3.39 × 10^−5^
	P2	−0.694	3.46 × 10^−6^
	P3	−0.552	3.87 × 10^−7^
	P4	−0.587	2.72 × 10^−7^
	P5	−0.585	9.75 × 10^−6^
Dispersant	D1	−0.639	2.48 × 10^−6^
	D2	−0.612	6.72 × 10^−6^
	D3	−0.587	2.72 × 10^−7^
	D4	−0.603	1.17 × 10^−6^
	D5	−0.692	7.46 × 10^−6^
	D6	−0.608	4.48 × 10^−5^

**Table 4:** Fitting Results of EIS of coated test panels made from different raw materials.

Sample		Rcoat/(Ω·cm^2^)
Emulsion	E1	3.36 × 10^2^
	E2	7.94 × 10^3^
	E3	5.43 × 10^3^
	E4	2.49 × 10^4^
	E5	8.93 × 10^3^
Pigment filler	P1	5.45 ×10^3^
	P2	6.38 × 10^3^
	P3	1.02 × 10^4^
	P4	2.49 × 10^4^
	P5	4.18 × 10^3^
Dispersant	D1	3.49 × 10^3^
	D2	4.00 × 10^3^
	D3	2.49 × 10^4^
	D4	2.98 × 10^3^
	D5	5.74 × 10^3^
	D6	3.69 × 10^3^

**Table 5:** Unidirectional tensile test results of samples N_0_, N_1_, N_2_.

Sample Name	Yield Load (KN)	Yield Strength (MPa)	Ultimate Load (KN)	Tensile Strength (MPa)	Bond Strength (MPa)
N_0_	70.39 ± 0.056	457.40 ± 0.364	96.66 ± 0.078	628.07 ± 0.507	19.11 ± 0.015
N_1_	71.62 ± 0.069	465.35 ± 0.483	93.07 ± 0.071	604.74 ± 0.461	18.40 ± 0.014
N_2_	73.6 ± 0.073	478.74 ± 0.473	96.86 ± 0.069	629.37 ± 0.403	19.15 ± 0.014

## Data Availability

Data are contained within the article.
